# Magnetic Waves vs. Electric Shocks: A Non-Inferiority Study of Magnetic Seizure Therapy and Electroconvulsive Therapy in Treatment-Resistant Depression

**DOI:** 10.3390/biomedicines11082150

**Published:** 2023-07-30

**Authors:** Helena Bellini, Eric Cretaz, Adriana Munhoz Carneiro, Pedro Henrique Rodrigues da Silva, Leonardo Afonso dos Santos, José Gallucci-Neto, André Russowsky Brunoni

**Affiliations:** 1 Service of Interdisciplinary Neuromodulation, Laboratory of Neurosciences (LIM-27), Instituto de Psiquiatria, Hospital das Clínicas, Faculdade de Medicina, Universidade de São Paulo, São Paulo 05403-010, Brazil; helena.bellini@hc.fm.usp.br (H.B.); ecretaz@uol.com.br (E.C.); adrianacarneiro01@gmail.com (A.M.C.); pedrojoanabrit@usp.br (P.H.R.d.S.); leonardo.afonso@hc.fm.usp.br (L.A.d.S.); galluccineto@gmail.com (J.G.-N.); 2 Service of Electroconvulsive Therapy, Instituto de Psiquiatria, Hospital das Clínicas, Faculdade de Medicina, Universidade de São Paulo, São Paulo 05403-010, Brazil

**Keywords:** depression, treatment-resistant depression, magnetic seizure therapy, electroconvulsive therapy, cognitive effects, magnetic resonance imaging, neurotrophic factors, neurophysiological biomarkers, non-inferiority trials, research design

## Abstract

Treatment-resistant depression (TRD), characterized by the failure to achieve symptomatic remission despite multiple pharmacotherapeutic treatments, poses a significant challenge for clinicians. Electroconvulsive therapy (ECT) is an effective but limited option due to its cognitive side effects. In this context, magnetic seizure therapy (MST) has emerged as a promising alternative, offering comparable antidepressant efficacy with better cognitive outcomes. However, the clinical outcomes and cognitive effects of MST require further investigation. This double-blinded, randomized, non-inferiority study aims to compare the efficacy, tolerability, cognitive adverse effects, and neurophysiological biomarkers of MST with bilateral ECT (BT ECT) in patients with TRD. This study will employ multimodal nuclear magnetic resonance imaging (MRI) and serum neurotrophic markers to gain insight into the neurobiological basis of seizure therapy. Additionally, neurophysiological biomarkers will be evaluated as secondary outcomes to predict the antidepressant and cognitive effects of both techniques. The study design, recruitment methods, ethical considerations, eligibility criteria, interventions, and blinding procedures are described. The expected outcomes will advance the field by offering a potential alternative to ECT with improved cognitive outcomes and a better understanding of the underlying pathophysiology of depression and antidepressant therapies.

## 1. Introduction

Depression is a prevalent global health issue, affecting more than 264 million people worldwide, according to the World Health Organization (WHO)**.** However, a significant proportion of individuals with depression do not achieve symptomatic remission even after multiple appropriate pharmacotherapeutic treatments. This condition is known as treatment-resistant depression (TRD) [1]. Managing TRD poses challenges for clinicians, as they must rule out misdiagnosis, comorbid disorders, inadequate therapeutic regimens, and non-compliance with treatment before considering additional strategies such as electroconvulsive therapy (ECT) [2].

Although ECT is an effective treatment for TRD, its cognitive side effects, such as impaired mnesic and non-mnesic abilities, limit its broader use [3]. While non-mnesic abilities impairment and anterograde amnesia generally improve within 15 days after the last ECT session, retrograde amnesia can persist for years, making it the most concerning cognitive side effect of ECT [4,5,6].

Recent years have witnessed the emergence of innovative treatments for mental disorders, including psychedelics and alternative agents. Some of these drugs, such as ketamine and dextromethorphan, target NMDA receptors and offer potential for more favorable clinical profiles. Nevertheless, further research is needed to understand their underlying mechanisms and develop effective treatments [7,8].

The lack of a clear understanding of the pathophysiology of depression and antidepressant therapies also hampers the development of neuromodulation techniques, including convulsive therapies. Although various strategies have been developed to mitigate ECT-induced cognitive effects, their results have been limited [9]. One possible explanation is that electrical stimulation diffuses throughout the brain, including deep structures crucial for memory retention, due to the high resistance offered by the skull and scalp [10].

Magnetic seizure therapy (MST) has emerged as an alternative convulsive therapy that overcomes the limitations of ECT. MST utilizes a magnetic field that encounters no resistance as it passes through the scalp and skull, enabling seizure induction without directly stimulating deeper brain structures [10]. A meta-analysis by Chen et al. [11] demonstrated that MST is as effective as ECT in terms of antidepressant efficacy, but with superior cognitive outcomes, including shorter recovery and reorientation times, as well as better performances in word recall, visual memory, and verbal fluency.

However, the clinical outcomes of MST, particularly its effects on cognitive performance, are not yet fully established [12]. Moreover, despite the availability of a more focal method of seizure induction, the therapeutic targets of MST remain poorly understood, limiting the development of individualized treatments [13]. To address these gaps, we propose a double-blinded, randomized, non-inferiority study to investigate the efficacy, tolerability, cognitive adverse effects, and neurophysiological biomarkers of MST compared to bilateral ECT (BT ECT) in patients with TRD. The study will employ multimodal nuclear magnetic resonance imaging (MRI) and analyze serum neurotrophic markers to gain a better understanding of the neurobiological basis of seizure therapy. Additionally, neurophysiological biomarkers will be evaluated as a secondary outcome to predict the antidepressant and cognitive effects of both techniques.

In this article, we present the design and recruitment process of the study, as well as the ethical considerations and eligibility criteria. Furthermore, we describe the interventions, including anesthesia and monitoring procedures for both ECT and MST. The blinding protocol and the frequency and number of sessions are also outlined. The study aims to provide valuable insights into the comparative effectiveness and safety of MST and ECT for TRD, contributing to the development of optimized treatment approaches for this challenging condition.

## 2. Materials and Methods

This section aims to outline the methodology employed in conducting the study, offering a comprehensive overview of the steps and procedures undertaken for data collection and analysis.

### 2.1. Study Design, Setting, and Recruitment

**Figure 1:**
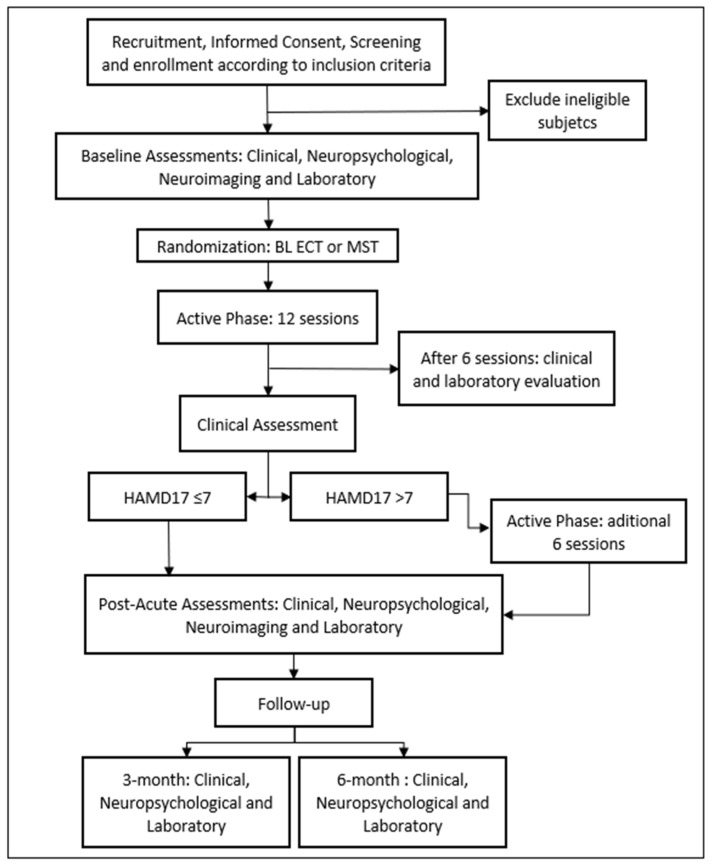
Study design (BL ECT, bilateral electroconvulsive therapy; MST, magnetic seizure therapy; HAMD17, Hamilton Depression Rating Scale, 17 items [17]).

The study involves a double-blinded, randomized, non-inferiority clinical trial [14] with two treatment arms (see Figure 1: study design) conducted at the Institute of Psychiatry, Hospital das Clínicas, São Paulo, Brazil.

Recruitment will be conducted through referrals from physicians in the ECT Service (mainly from the Institute of Psychiatry), self-referral via university community advertisements (e.g., leaflets and email), and advertisements on the website (www.sin.org.br), Google, and social media platforms such as Facebook.

After the recruitment and selection process, participants will undergo a baseline assessment, which includes medical evaluation, neuropsychological assessment, laboratory tests, and cranial MRI. They will then be divided into two groups (ECT and MST). The study will utilize a 1:1 controlled randomization model with blocks of random sizes and lengths generated through www.sealedenvelope.com.

Generally, during the acute phase of ECT, the frequency of treatment sessions varies from one to three times per week. Studies have indicated that ECT conducted twice a week shows comparable efficacy to ECT performed three times a week, while requiring a reduced number of treatments. Additionally, there appears to be greater efficacy of ECT applied three times a week compared to the weekly application schedule [15]. Regarding cognitive functioning, the more frequent the sessions, the greater the cognitive impairments [16]. Consequently, we have opted to conduct ECT or MST sessions during the active phase with a frequency of twice a week on non-consecutive days.

Initially, patients will receive a total of 12 sessions, including the first successful titration session. If the first titration session fails to induce adequate seizures, it will not be considered as part of the total number of sessions. After the 12th session, a clinical assessment will be performed. If the patient meets the criteria for remission, defined as a score of 7 or less on the 17-item Hamilton Depression Scale (HAMD17) [17], the sessions will be discontinued, and the patient will undergo a neuropsychological evaluation. If the patient does not achieve remission, they will receive an additional 6 sessions, bringing the total to 18, and will be re-evaluated and undergo a neuropsychological assessment. In this case, regardless of the response or remission, treatment will be discontinued after the 18th session. This is because evidence suggests that the number of sessions required to achieve response and/or remission typically ranges from approximately 6 to 15 sessions [18].

Following the final session, participants will undergo a comprehensive evaluation, including medical assessment, neuropsychological evaluation, laboratory tests, and cranial MRI. Finally, patients will be followed up for 6 months after completing the intervention, with re-evaluations at 3 and 6 months.

### 2.2. Ethical Considerations

The Local and National Ethics Committees approved the clinical trial study, which adheres to the Helsinki guidelines and their subsequent amendments. All recruited volunteers will undergo medical screening, during which the professional will explain the objectives, interventions, procedures, and potential risks of the trial. They will also receive a detailed consent form, and any questions will be answered. Written informed consent is required before starting the eligibility assessment, and patients can withdraw from participation at any time during the study. Patients who have an indication for ECT will be enrolled, as the risk of complications from ECT is low [16]. Patients will be closely monitored, and if their depression worsens, they will be discontinued from the study and treated accordingly. Participation may offer benefits such as psychiatric and psychological assessments, improvement in depression, standard care, and appropriate referrals. MRI safety criteria will be followed, and patients with contraindications will be excluded. The MRI is safe, and participants will not receive sedation, anesthesia, or contrast agents. Participants will receive medical feedback from a certified radiologist regarding their brain scan, which is a benefit.

### 2.3. Eligibility Criteria

Trained psychiatrists/psychologists will review the diagnosis of depression using the Brazilian version of the Mini International Neuropsychiatric Interview 5.0.0 (MINI) [19], which was developed based on the *Diagnostic and Statistical Manual of Mental Disorders*, 4th edition (DSM-IV) and International Classification of Diseases and Related Health Problems, 10th revision (ICD-10). 

Inclusion criteria are: (a) age between 18 and 60 years; (b) diagnosis of major depressive disorder or depressive episode in bipolar disorder according to DSM-IV; (c) baseline HAMD17 score ≥17; (d) current TDR episode; (e) appropriate clinical condition for receiving ECT as assessed by a psychiatrist and an anesthesiologist; and (f) meeting transcranial magnetic stimulation (TMS) safety criteria [20]. In the definition of TRD for major depressive disorder, the utilization of a minimum of two prior treatment failures will be considered. Conversely, for TRD in bipolar depression, only one prior treatment failure will be required. In both cases, there must be confirmation of prior adequate dose and duration, based on clinical criteria evaluated using the Antidepressant Treatment History Form (ATHF) [21,22].

Exclusion criteria are: (a) pregnancy; (b) psychiatric comorbidity (except mild anxiety disorders); (c) depressive symptoms better explained by other clinical conditions or psychiatric disorders; (d) decompensated clinical and neurological conditions or serious sequelae of previous medical conditions; (e) use of psychoactive substances in the last 6 months (except nicotine); (f) use of monoamine oxidase inhibitors in the last 6 months; (g) application of ECT or another neuromodulation technique in the last 12 months; (h) contraindications for ECT or MRI; (i) relevant deviations in clinical laboratory parameters; or (j) inability to consent.

Discontinuation criteria are: (a) worsening depression; (b) development of suicidal ideation or suicide attempt; (c) withdraw consent; (d) development of (hypo)manic symptoms; (e) inability to induce seizures lasting at least 15 s after adjusting parameters and medications during the titration phase; (f) missing two consecutive treatment sessions; or (g) changes in drug treatment without the previous authorization.

Patients will need to have a stable drug regimen for at least 2 months before the first clinical and neuroimaging evaluations. Medications that alter the seizure threshold, such as benzodiazepines and mood stabilizers with anticonvulsant action, will be closely monitored. If necessary, the team will evaluate the possibility of reducing the doses of these medications before the second titration session.

These eligibility criteria are consistent with the criteria employed in important ECT trials conducted in recent years [23,24,25] and another comparative study protocol between ECT and MST [26].

### 2.4. Interventions

#### 2.4.1. Anesthesia and Monitoring

We have developed anesthesia protocols based on the most recent evidence available in the literature [27] and the availability of resources in our setting. Basically, trained anesthesiologists with ECT experience will administer general anesthesia using etomidate (0.5 mg/Kg), succinylcholine (0.6 mg/Kg), and atropine (0.5–1 mg) to prevent cardiac arrhythmias. Patients will be ventilated with a 100% oxygen mask, and cardiovascular parameters will be monitored throughout the procedure. The seizure threshold (ST), which is the minimum energy required for a generalized tonic-clonic seizure, will be determined at the beginning of the study using specific protocols for each technique, which will be described below. Seizures will be considered adequate if they result in generalized tonic-clonic activity lasting more than 20 s of motor or electroencephalographic (EEG) activity, according to the guidelines of the American Psychiatric Association (1990) [28]. The EEG monitor of the Magventure (Farum, Denmark) MagPro MST device will be used for this purpose, which allows for digital storage of tracings for patients undergoing ECT and MST. 

#### 2.4.2. ECT

The ECT sessions will be conducted with the Thymatron System IV (Somatics LCC, Venice, FL, USA). The pulse width and amperage will be fixed at 0.5 ms and 0.9 A, respectively, while other parameters such as pulse frequency will be automatically determined by the device based on the selection of the operator. The electrodes will be positioned bilaterally in the temporal region, as this is associated with a more effective and rapid response to depressive symptoms [16]. The electrodes will receive conductive gel and will be positioned about four centimeters perpendicularly above the midpoint of an imaginary line between the lateral epicanthal fold and the tragus. These parameters are in accordance with the local practice of the ECT Service of the Institute of Psychiatry.

To determine the electrical load, a dose titration process will be conducted during the first session of each individual [29]. This process will begin with a 5% load (equivalent to 25 mC). If this stimulus is not effective in triggering an adequate generalized convulsive crisis, the load will be doubled and the stimulus repeated until an effective crisis is triggered or a maximum threshold of 3 to 4 attempts is reached, depending on the assessment of the psychiatrist and the anesthetist according to physiological parameters (blood pressure, oxygen saturation, heart rate) and depth of sedation. If a seizure is not initiated in the first session, a new titration session will be scheduled, and medication will be reviewed.

The second titration session will repeat the parameters used in the last attempt and will follow the same procedures described above. If seizures are still not initiated in the second session, the patient will be excluded from the study. In subsequent sessions after the titration, the load used will be two and a half times, rounding down when necessary. If the patient has seizures of less than 20 s duration, the load will be increased by 100% in the next session until the seizures are effective or the maximum load of the device is reached.

#### 2.4.3. MST

The MagVenture MagPro MST with Twincoil (Magventure, Farum, Denmark) will be used for MST sessions. The procedure will follow the same guidelines as ECT sessions regarding general anesthesia, oxygenation, motor activity monitoring, EEG, and postictal recovery. The Twin Coil will be centered over point Fz of the 10–20 system using an EEG cap to mark the correct point on the patient’s scalp.

The stimulation will be delivered with an amplitude of 100%, 100 Hz. In the first session, a titration of the convulsive threshold will be performed with a 2 s stimulus (200 pulses). If an adequate seizure is not triggered, the stimulus will be increased by two seconds (200 pulses) until a seizure is triggered or a maximum of 3 to 4 attempts is reached. If a seizure is not initiated in the first session, a new titration session will be scheduled. Medication will be reviewed and, if possible, the dose of benzodiazepines will be reduced depending on the opinion of the performing psychiatrist. The second titration session will repeat the same process as the first, with the parameters used in the last attempt. The maximum limit is set at 10 s of stimulation or until the coil temperature exceeds the safety limit. If no seizure is triggered in the second session, the patient will be excluded from the study. In subsequent sessions, the stimulus used will be 4 s (400 pulses) higher than the titration session, up to a maximum of 1000 pulses. If the patient has seizures of less than 20 s duration, the number of pulses will be increased by 200 pulses in the next session, until the seizures are effective or the limit of 1000 pulses is reached. These parameters were determined based on our previous experience [30].

#### 2.4.4. Blinding

Participants will not be informed of the device used for their seizure therapy until the end of the trial. Patients receiving ECT will have their Fz point marked with an EEG cap, and during the procedure, the MST device will be activated due to its distinct noise. Similarly, patients receiving MST will have conductive gel applied to their temples, as in ECT application. The application room curtains will be drawn to prevent other patients from observing the procedures. The staff responsible for the procedures will not carry out the clinical and cognitive evaluations. Trained professionals will be assigned to perform these tasks. Additionally, nursing staff will not provide patients with any information about the procedures performed.

After the end of the clinical trial, patients will be asked to guess the group they were assigned to, as well as their confidence in their guess, following the protocol of our previous studies [31]. Survival analysis will be conducted using relapse as the event, and Cox ratios will be used to evaluate the impact of clinical and demographic variables on the outcome.

### 2.5. Clinical and Cognitive Measures

All data will be securely stored in an electronic data capture system called Research Electronic Data Capture (REDCap) (https://redcapbrasil.com.br/).

The validated Portuguese versions of the clinical and sociodemographic assessment scales will be applied during screening, at baseline, in the active phase (after the 6th session) and during follow-up (one week, three months, and six months after the last session), as shown in Table 1. The co-primary outcome scales are the HAMD17 [17] and the Autobiographical Memory Task (AMT) [32]. Remission will be defined as a final HAMD17 score less than or equal to 7 points, and a 50% reduction in score compared to baseline assessment will be considered a response. 

Experienced neuropsychologists will perform a cognitive assessment, which will last approximately 120 min, at baseline and at follow-up (one week and six months after the last session), as shown in Table 2.

### 2.6. Safety Monitoring

Pre-anesthetic and dental evaluation will be performed before the start of the sessions. Adverse events and safety will be monitored and recorded at every study visit. To assess any adverse effects during and after the active phase of treatment, we will use the Non-Cognitive Adverse Effects Questionnaire for ECT/MST [51], which evaluates symptoms such as headaches, muscle aches, nausea, prolonged seizures, and severe events. We will also use the Young Mania Rating Scale (YMRS) [52] to assess the occurrence of (hypo)manic induction. Additionally, the Orientation Scale, a 10-item questionnaire, will be administered 30 min after each seizure treatment. Sobin et al. [53] found a positive correlation between postictal reorientation time and retrograde amnesia. If any harm occurs as a result of study participation, appropriate care will be provided to participants.

### 2.7. Neuroimaging

Baseline and post-intervention images will be acquired using a 3.0 T device (Achieva, Phillips, Amsterdam, The Netherlands) located at the Radiology Institute of the Hospital das Clínicas of the Faculty of Medicine of the University of São Paulo (USP). The device is equipped with a Quasar Dual gradient system (maximum intensity of 80 mT/m and variation rate of up to 200 mT/m/ms) and a dedicated 8-channel skull coil with availability of the SENSE technique. Paramagnetic contrast medium will not be administered, and head position will be secured with foam padding to minimize movement. During the approximately 60 min procedure, cardiac and respiratory monitoring will be performed to eliminate possible physiological noises from these sources in the image pre-processing stages [54]. Patients will be instructed to keep their eyes closed, not move, and think as little as possible during the exam.

The anatomical dataset will include T1-weighted structural MRI sequences acquired for voxel-based morphometry (VBM) analysis. Group comparison statistics for each voxel of brain volume can be performed using VBM, allowing the quantification of the local volume of white or gray matter and automated extraction of regions of interest (ROIs) [55]. The assessment of cortical thickness in ROIs will also be performed. Additionally, diffusion-weighted images (DWIs) will be obtained to measure the anisotropy/isotropy index and the diffusivity of water molecules and to evaluate structural connectivity. Resting-state functional MRI (rsfMRI) based on the blood-oxygen-level-dependent (BOLD) effect will also be acquired to assess functional connectivity.

The volumetric weighted 3D T1 images will be recorded using the following parameters: repetition time (TR) = 7.0 ms, echo time (TE) = 3.2 ms, angle of inversion (FA) = 8°, direction 1.5, field of view (FOV) = 240 × 240 mm, matrix = 240 × 240 pixels, 180 slices of 1 mm each, no gap, producing a voxel size of 1 mm × 1 mm × 1 mm. A weighted FLAIR sequence high-resolution T2 will also be collected to increase accuracy for automatic segmentation of structures. These images can be used in the following configurations: TE = 120 ms, TR = 2800 ms, 60 axial slices (thickness = 3 mm), AF = 8°, FOV = 230 mm × 230 mm.

The DWI will be collected using an EPI sequence single-shot (echo planar imaging) with 64 directions of non-collinear gradients (sampled across the sphere) through a b-shell (b = 1000 s/mm^2^) plus 08 volumes B0 (no broadcast) interleaved. The following parameters will be defined: TE = 100 ms, TR = 8801 ms, angle of inclination = 90°, FOV = 256 mm × 256 mm, acquisition matrix = 256 × 256, 64 slices, slice thickness = 2.0 mm (no gap between slices), yielding a size of 2 mm × 2 mm × 2 mm voxel. 

We will acquire rsfMRI using the EPI T2*-weighted sequence, with the following parameters: SENSE 2, TR = 2200 ms, TE = 28 ms, 3 mm slice thickness, 3 mm isometric voxel size, 0.3 mm slice interval, FOV of 240 × 240 × 125, and a frame size of 80 × 80 matrix. This sequence will result in 38 slices/volume and a total of 300 volumes. To minimize motion effects, we will use stabilizer dynamics. 

We will store the processed images in DICOM format to facilitate data sharing [56], anonymized by removing facial features, and transfer them to our dedicated workstation. We will perform a quality check, pre-process the images, and analyze them with different software, including FSL 4.1 (FMRIB, Oxford, UK), Freesurfer 7.4.1 (Computational Neuroimaging Laboratory, Harvard Medical School, Cambridge, MA, USA), SPM 12 (Wellcome Department of Imaging Neuroscience, London, UK), and internal scripts based on image type (e.g., structural, functional).

Cortical brain volume, area, and cortical thickness will be quantified for the dorsal prefrontal cortex (DLPFC) and subgenual anterior cingulate cortex (sgACC) using the Glasser parcellation [57]. Subcortical brain volume will also be quantified for the hippocampus and amygdalae. These regions were selected based on previous studies [58,59,60]. Both subcortical and cortical volumes will be divided by the total intracranial volume (TIV) to control for differences in brain size among participants. Structural and functional connectivity will be carried out using these ROIs as seeds. 

Statistical analyses will be performed using R Version 4.1.2. We will use linear mixed models with a three-way interaction between the fixed predictors MRI measure, time (pre-treatment and post-treatment), and treatment (ECT group vs. MST group). Participants will be included as random effects. All results will be considered significant at a p threshold of 0.05. Outcomes will be the HAMD-17 scores. To each three-way significant interaction, we will perform two-way interaction between the fixed predictors MRI measure and time separately to each group. The same analysis will be performed within each treatment group. We will also explore associations between baseline MRI-based biomarkers and ECT and/or MST effectiveness, evaluated with the changes in depression scores measured by HAMD-17 using linear mixed models. All the reported values will be based on the three-way or two-way interaction of the fixed variables.

### 2.8. Seric Biomarkers

Venous blood collections will be performed in the morning, fulfilling a minimum fasting period of 8 h comprising the following events:

- C1 (Collection 1): Performed on the day corresponding to the first session, prior to the procedure, taking advantage of the need for venipuncture. This will be considered the baseline sample;
- C2 (Collection 2): performed prior to 6th session;
- C3 (Collection 3): performed prior to 12th session;
- C4 (Collection 4): performed prior to 18th session (if any);
- C5 (Collection 5): performed 12 weeks after the start of the treatment;
- C6 (Collection 6): performed 24 weeks after starting treatment, coinciding with the 3-month medical evaluation.

Two 4 mL tubes of venous blood samples with anticoagulant (EDTA) will be collected before each event to prevent biochemical alterations related to the convulsive crisis. The collections will be performed at the ECT Service and immediately transported to the Neuroscience Laboratory LIM-27 for proper storage until the biochemical analysis is conducted. Post-treatment collections (C5, C6, and C7) will be scheduled with the staff and patient at the Neuroscience Laboratory LIM-27 after an 8 h fast. Brain-derived neurotrophic factor (BDNF) will be evaluated in the plasma using an immunofluorescence ELISA technique, which is highly specific for this type of evaluation. This technique has been shown to be more appropriate for state-dependent assessment of BDNF levels than other methods [61].

Our primary objective is to explore potential associations between changes in plasma BDNF levels and variations in depressive symptom scores and treatment response, as well as other clinical and neuroimaging outcomes (such as the variation in volume of each ROI). Additionally, we intend to compare the differences in plasma BDNF variation over time in patients undergoing ECT or MST. Moreover, we will evaluate the discrepancies in BDNF levels between patients who respond positively to treatment and those who do not, in each treatment arm.

Based on previous studies [62,63,64,65], our hypothesis is that there will be a progressive increase in plasma BDNF levels, especially in the subgroup of patients with lower BDNF levels at baseline. We also hypothesize that there will be a positive correlation between improvement in depressive symptoms and an increase in BDNF, but its significance will be observed specifically in relation to plasma BDNF collected at time points C5 and C6. Additionally, we believe that higher baseline plasma BDNF levels may be associated with higher rates of treatment response and remission and, possibly, better cognitive outcomes. Furthermore, there will be a similar increase in plasma BDNF levels in patients who undergo ECT when compared to those who receive MST.

### 2.9. Statistical Analysis

Statistical analyses will be performed in R. The results will be considered statistically significant only if both conditions are met, that is, non-inferiority for the depression outcome, under unilateral *p* < 0.025, and superiority for the cognitive outcome, under *p* bilateral < 0.05. The following groups will be considered: experimental (E), corresponding to MST, and control/comparison (C), corresponding to ECT.

For the co-primary outcome depression (HAMD17), non-inferiority will be assessed by calculating the difference between the means of groups E and C and its 95% confidence interval at the end of the 12th session, which will then be compared to the NI margin (Ha: C-E < NI). Analyses will be performed on modified intent-to-treat (ITT) samples (at least 8 sessions of ECT/MST) and per-protocol (PP).

For the co-primary cognitive outcome (AMT [32]), we will perform linear hierarchical model analyses using a first-order regressive covariance structure, which includes all observed variables without the need to enter data for missing values. The dependent variable is the AMT score [32], the independent variables are time (all observations through week 6) and group. We will test the statistical significance of this interaction according to our superiority hypotheses controlled for age, years of schooling, and treatment-resistant depression (Ha: C-E < 0). Analyses will be performed on the ITT and PP sample.

The same linear hierarchical models will be used for all continuous secondary outcomes. We will assess response and sustained remission, that is, a clinical improvement of symptoms ≥50% (response) and a HAMD17 score ≤7 (remission) at a given point in the study, which will be maintained until the outcome. The trajectories of response and sustained remission between groups will be compared using Kaplan–Meier survival analysis. Response and remission will be compared considering non-inferiority margins of 20% and 15%, respectively. The choice of a 15% non-inferiority margin was based on the consideration that achieving a remission rate of 35% holds clinical relevance in this population with difficult-to-treat depression. Furthermore, this remission rate surpasses those achieved by more conventional and less invasive treatment options for TDR, such as repetitive TMS (rTMS) with an approximate remission rate of 20% [66] and antidepressants with a remission rate of 14% [1].

We will define adverse events as events with intensity greater than “mild” and at least “remotely” associated with the intervention, as carried out in our previous studies [31]. The presence of adverse events and (hypo)manic upset will be compared between groups E and C using the chi-square test. This test will also be used to assess whether allocation group guessing occurred beyond chance.

Regarding the BDNF data, each collection during the follow-up will be individually analyzed in relation to the baseline values using Student’s *t*-test. The same analysis will be conducted to assess the differences between the treatment groups (ECT x MST) at each endpoint. Additionally, analysis of variance (ANOVA) will be used to evaluate potential differences in the means of plasma BDNF at different collection time points. A significance level of 5% will be adopted for all tests.

### 2.10. Sample Size Calculation

The software programs G * Power 3 [67] from Universität Düsseldorf, Germany, and Stata (Statacorp, College Station, TX, USA) were used to conduct calculations. The sample size was determined using parameters of α = 0.025 (one-tailed), β = 0.2, and a standard deviation of 9.5. The margin of non-inferiority (NI) was determined based on a combination of statistical reasoning and clinical judgment [68]. The lower bound of the confidence interval from a meta-analysis comparing ECT vs. sham was used to establish the NI [69]. Using a meta-analysis by the UK ECT Review Group [16], the difference in HAMD17 scores between ECT and placebo was 9.7 points (95% CI 5.7 to 13.5), with an NI value of 5.7. Despite the large margin, this NI value was deemed acceptable due to the cognitive effects of bitemporal ECT (the control comparison). Thus, MST would be a reasonable non-inferior intervention, as cognitive superiority would also need to be demonstrated. The sample size required was 88 patients, with an additional enrollment of 10% (total of 100 patients, 50 per arm) to account for attrition. For the superiority analysis, we used data from an unpublished clinical trial of 75 patients that compared the efficacy and cognitive outcomes of MST vs. ECT (personal communication from Prof. McClintock). The effect size of AMT [32] after 12 treatment sessions was approximately 1.31, requiring a sample size of 26 patients to demonstrate a difference. Thus, the larger sample size from the clinical outcome study was used for the NI calculation.

## 3. Discussion

ECT is an effective alternative for treating TRD, albeit with limitations attributed to cognitive side effects [18]. Recent advancements in therapeutic modalities have introduced alternative agents and neuromodulation techniques, such as MST. By utilizing a magnetic field, MST offers a potential solution to the cognitive side effects associated with ECT by inducing seizures without directly stimulating deeper brain structures [70]. The objective of this study is to present a double-blinded, randomized, non-inferiority trial that compares the efficacy, tolerability, cognitive adverse effects, and neurophysiological biomarkers of MST to BT ECT in patients diagnosed with treatment-resistant unipolar or bipolar depression.

The primary outcome measure will be the reduction in depressive symptoms, assessed using the HAMD scale. Secondary outcome measures will include response rates, remission rates, and cognitive functioning assessed through comprehensive neuropsychological testing. Simultaneously, the evaluated biomarkers include multimodal MRI and serum BDNF levels. Evaluation of outcomes will be conducted at baseline, during the active treatment phase, and throughout a 6-month follow-up period.

Building upon previous research, the proposed protocol explores the potential of utilizing MST as an alternative form of convulsive therapy for TDR. This represents a significant advancement in the field.

The initial experiments with MST demonstrated its feasibility, tolerability, and effectiveness in both non-human primates and humans [71,72]. Shortly after, a double-blind randomized trial comparing the side effects of ECT and MST in patients diagnosed with major depression reported fewer subjective side effects and lower rates of disorientation, inattention, retrograde amnesia, and impairment of verbal fluency in the immediate post-ictal period for patients undergoing MST [70]. Since then, several studies have investigated the efficacy of MST for the treatment of depression, particularly when compared to ECT [73,74,75,76]. In general, recent studies provide support for the hypothesis that MST, when compared to ECT, exhibits non-inferiority in terms of antidepressant effects [77,78,79] and demonstrates a more favorable cognitive profile [73,74,75,77,80,81]. 

Despite the promising evidence, it is important to note that the evidence in favor of or against MST is not sufficiently robust, and further scientific evidence is required to establish its therapeutic potential. A meta-analysis conducted by Chen et al. [11] highlighted the scarcity of studies and the heterogeneity in methodologies employed, indicating the need for more research with larger sample sizes and standardized protocols. Moreover, they also emphasized that the majority of previous studies (1) primarily focused on memory when assessing cognition, neglecting other cognitive abilities; (2) lacked follow-up evaluations, thus obscuring the long-term clinical effects; and (3) predominantly utilized RUL ECT, which may be associated with lower efficacy compared to BT ECT [16], potentially overestimating the comparative antidepressant therapeutic effect of MST.

Through the implementation of a rigorous methodology to ensure validity and reliability, our study aims to address existing gaps in the literature. By extending the follow-up period beyond the active phase of the study, we seek to gain insights into the sustained benefits and relapse rates associated with MST. Furthermore, we intend to assess the effects of MST on various cognitive dimensions beyond memory assessment, providing a comprehensive understanding of its cognitive impact. Additionally, we plan to compare MST with BT ECT, taking into consideration the more favorable antidepressant efficacy profile of BT ECT. This comparative analysis will contribute to a comprehensive understanding of the therapeutic potential and effectiveness of MST in real-world clinical settings.

We anticipate that our study will provide evidence supporting the non-inferiority of MST compared to BT ECT in reducing depressive symptoms among patients with TRD. Moreover, we hope to demonstrate that MST exhibits a more favorable cognitive profile, particularly with respect to memory function, when compared to BT ECT. The availability of a viable and effective alternative convulsive therapy may improve access to treatment for drug-resistant patients who currently face severe and debilitating symptoms due to concerns or apprehensions about ECT-induced side effects.

The second significant potential of the proposed protocol, building upon prior research, pertains to the exploration of the neurobiological underpinnings of convulsive therapy. While MST theoretically provides greater control over the induction and spread of seizures, individualized treatments require knowledge of the specific biological targets to stimulate. However, the neurobiological basis of convulsive therapy and the relationship between its antidepressant and cognitive effects are not fully understood. Existing evidence primarily comes from studies on ECT, which have limitations such as small sample sizes, methodological diversity, and inconclusive results [82,83].

To the best of our knowledge, there have been fewer than five studies to date investigating the neurobiological underpinnings of MST. For example, Cycowicz et al. [84] conducted a study with rhesus macaque monkeys, randomizing them to receive ECS (form of ECT applied to animals in research studies), MST, or simulated ECT over a period of six weeks. EEG analysis revealed that MST induced lower ictal EEG power compared to ECS, primarily due to increased activation in the alpha, beta, and theta bands. Delta activity did not show significant differences between the techniques. These findings suggest that superficial cortical regions may be anatomically associated with the antidepressant effects of convulsive therapies. Additionally, the intensity of ECS stimulation influenced the expression of higher frequency bands but not delta activity. It is known that increasing the intensity of ECT above the seizure threshold leads to cognitive side effects without enhancing efficacy. This suggests that the side effects of convulsive therapy may be partially related to the stimulation of deeper brain regions. 

One of the challenges in investigating the neurobiological basis of convulsive therapy pertains to the fact that these techniques exhibit therapeutic effects on various pathologies that are conceptually distinct. Their mechanism of action involves different levels of the biological cascade, including cell receptors, gene expression, protein synthesis, neuroplasticity, and connectivity between different brain circuits. Consequently, some of the biomarkers considered crucial may only be epiphenomena in relation to the antidepressant response. In this sense, the main question to be addressed is the extent of neuroanatomical overlap between the clinical effects of both modalities of convulsive treatment. This is one of the innovations of our protocol, which provides an opportunity to advance our understanding of the mechanisms of action of convulsive therapy in the treatment of disorders such as depression.

Another significant innovation of our protocol is the combination of different biomarkers, including multimodal neuroimaging. MRI images can be used to measure phenotypic variations in specific brain circuits that represent a unique interaction between genes and the environment and are associated with specific behavioral changes [85]. Most studies that have analyzed these biomarkers in ECT studies have only utilized a single MRI modality [86]. The use of a combination of different MRI parameters, sensitive to complementary tissue characteristics, can provide a basis for the construction of more precise endophenotypes, facilitating the identification of patient groups more likely to respond to specific interventions [87], contributing to the development of precision psychiatric clinical practice.

Comprehending the neurobiological foundations of convulsive therapy holds the potential to enable precise targeting of circuits implicated in specific symptoms while preserving cognitive functions, thereby reducing stigma and enhancing treatment accessibility. Furthermore, this knowledge can provide guidance for future research endeavors and drive advancements in the development of current therapeutic devices. Moreover, unraveling these biomarkers can contribute to our understanding of the intricate pathophysiology of depression and shed light on the neural mechanisms underlying the antidepressant effects of alternative treatment modalities, including pharmacotherapy.

Despite the clinical significance and robustness of this protocol, it is crucial to address several limitations. Firstly, the absence of a placebo control group hinders the ability to differentiate the specific effects of the interventions from placebo effects. A placebo control group would provide valuable insights and enhance the internal validity of the study. Secondly, the recruitment being limited to a single institution in Brazil raises concerns about the generalizability of the findings. It is important to recognize that the utilization of ECT in Brazil is typically limited to severe cases due to resource constraints, potentially limiting the applicability of the results to broader populations. Furthermore, the lack of assessment of serum drug levels represents a limitation in confirming the presence of pharmacological resistance in the study participants.

Additionally, the relatively short duration of follow-up restricts the ability to comprehensively evaluate the sustained effects of the interventions. A longer-term assessment would offer a more complete understanding of the long-lasting impacts. Moreover, the reliance on self-reported measures introduces the potential for recall bias and social desirability bias, which may affect the accuracy of the collected data. It is crucial to consider these limitations and interpret the findings in light of these potential biases. Lastly, the study population consists of a specific demographic group, and caution should be exercised when extrapolating the results to other populations with different characteristics, highlighting the need for further research in diverse populations to ensure broader applicability.

On the other hand, our protocol exhibits several strengths that enhance its scientific value. Firstly, it introduces a novel treatment option, MST, which has demonstrated promising safety and efficacy in preliminary evidence. Secondly, the protocol adopts a double-blinded, randomized, non-inferiority study design, ensuring a rigorous approach to comparing the efficacy and safety of MST and ECT. This design enhances the internal validity of the study. Thirdly, the protocol encompasses a comprehensive set of outcome measures, including assessment of depressive symptoms, cognitive performance, neurophysiological biomarkers, and neurotrophic markers. This multidimensional assessment provides a more comprehensive understanding of the effects of the interventions. Fourthly, the protocol incorporates multimodal nuclear magnetic resonance imaging (MRI) and analysis of serum neurotrophic markers to investigate the neurobiological basis of seizure therapy. This neurobiological investigation contributes to a deeper understanding of the underlying mechanisms of treatment-resistant depression (TRD) and the effects of the interventions. Fifthly, the evaluation of neurophysiological biomarkers as secondary outcomes has the potential to predict the antidepressant and cognitive effects of both techniques, paving the way for the development of individualized treatment approaches in the future. Sixthly, the protocol includes intensive clinical and neurocognitive assessments before, during, and after treatment, ensuring close monitoring of patient progress and ensuring their safety throughout the study. Lastly, the protocol incorporates longitudinal follow-up assessments post-treatment to monitor the long-term outcomes of patients, providing valuable insights into the durability of treatment effects. Collectively, these strengths contribute to the scientific robustness of our protocol and enhance its potential to generate valuable insights into the effectiveness and mechanisms of action of MST and ECT in the treatment of TRD.

In general, our results can contribute to the development of new strategies for the treatment of depression. However, it is important to note that the translation of research protocols to clinical practice requires further validation, replication, and consideration of real-world challenges and constraints. The results of this study would be an important step towards establishing MST as a safe and effective treatment option for TRD, but additional research and practical implementation considerations are necessary before widespread adoption in clinical settings.

## 4. Conclusions

This proposed study aims to contribute to the growing body of research on MST as an alternative to BT ECT for the treatment of TRD. By comparing the efficacy, tolerability, cognitive adverse effects, and neurophysiological biomarkers of MST and BT ECT, we hope to provide valuable insights that can inform clinical decision making and improve the quality of care for these patients.

## Figures and Tables

**Table 1:** Clinical measures.

Measure	Study Period					
	Prior to Acute Phase		Active Phase *	Follow-Up		
	Screening	Baseline		Post-Acute **	3 Months	6 Months
Inform Consent	x					
Demographics Form	x					
ABEP		x				
Medical History Form	x					
ATHF	x					
MINI	x					
QCT		x				
BPI		x				
HAMD17	x	x	x	x	x	x
MADRS		x	x	x	x	x
CGI		x	x	x	x	x
BDI-II		x	x	x	x	
DTS		x	x	x	x	x
BSI		x	x	x	x	x
WHOQOL		x		x	x	x

ABEP, Brazil Criterion of the Brazilian Association of Research Companies 2016 [33]; ATHF, Antidepressant Treatment History Form [22]; MINI, Mini International Neuropsychiatric Interview [19]; QCT, Questionnaire on Childhood Trauma [34]; BPI, Borderline Personality Inventory [35]; HAMD17, Hamilton Depression Rating Scale, 17 items [17]; MADRS, Montgomery–Asberg Scale [36]; CGI, Clinical Global Impression Severity (CGI-S) and Clinical Global Impression Improvement (CGI-I) scales (we will apply both the CGI-S and CGI-I) [37]; BDI-II, Beck Depression Inventory [38]; DTS, Depressive Thoughts Scale [39]; BSI, Beck Suicidal Ideation Scale [40]; WHOQOL, WHO quality of life questionnaire [41]; * after the 6th session; ** one week after the last session (12th or 18th, if applicable).

**Table 2:** Neurocognitive measures.

Domain	Measures	Study Period		
		Baseline	Post-Acute	6 Months
Estimated Intelligence Quotient (IQ)	WASI (Vocabulary, Matrix)	x	x	x
Attention/executive functioning; cognitive flexibility and cognitive processing speed	TMT (A & B) and FDT	x	x	x
Verbal and visual episodic memory	RAVLT and BVMT-R	x	x	x
Semantic memory and verbal fluency	FAS	x	x	x
Working memory	Foward and Reverse DGS (WAIS-III)	x	x	x
Cognitive Screening	MoCA	x	x	x
Autobiographical Memory	AMT	x	x	x
Prospective and Retrospective Memory	PRMQ	x	x	x

WASI, The Wechsler Abbreviated Scale of Intelligence [42]; TMT (A and B), Trail Making Test versions A and B [43]; FDT, Five Digit Test [44]; RAVLT, Rey Auditory Verbal Learning Test [45]; BVMT-R, Revised Brief Visuospatial Memory Test [46]; FAS, Verbal Fluency [47]; DGS—WAIS-III, Digit Span—The Wechsler Adult Intelligence Scale, Third Edition-III [48]; MoCA, Montreal Cognitive Assessment [49]; AMT, Autobiographical Memory Task [32]; PRMQ, Prospective and Retrospective Memory Questionnaire [50].

## Data Availability

Not applicable.
